# Radical Cheilectomy as an Alternative to Arthrodesis for Hallux Rigidus

**DOI:** 10.7759/cureus.9453

**Published:** 2020-07-29

**Authors:** Steven R Edwards, Andrew C Kingsford

**Affiliations:** 1 Surgery, Australasian College of Podiatric Surgeons, Melbourne, AUS; 2 Podiatry, La Trobe University, Bundoora, AUS

**Keywords:** hallux disorders, joint preservation surgery, arthroplasty, foot surgery techniques, great toe, forefoot

## Abstract

Hallux rigidus (HR) is a painful condition involving osteoarthrosis and reduced range of motion of the first metatarsophalangeal joint (MTPJ). It is associated with significant morbidity and reduced quality of life. We report a case of a 42-year-old female who had been referred to our surgical clinic regarding the progressively worsening chronic pain, stiffness and long-term shoe-fitting difficulties associated with her right HR pathology. Her vocational duties within the fashion industry necessitated the use of high heeled court-style shoes, and thus she maintained a preference for a procedure that would facilitate normal joint range of motion so that she could continue to wear this type of footwear. We performed a variation to a traditional cheilectomy procedure involving radical remodelling of the first metatarsal head to allow for up to 90 degrees of intraoperative dorsiflexion. The patient reported reduced pain and increased function up until her discharge at 12 weeks postoperatively. A radical cheilectomy may provide acceptable pain relief and improved joint function in patients with end-stage HR who decline the option of arthrodesis.

## Introduction

Hallux rigidus (HR) involves osteoarthrosis of the first metatarsophalangeal joint (MTPJ). The exact aetiology of HR is unclear, yet is most often attributed to a historic injury, rheumatic disease or foot postural factors [1]. It is associated with significant morbidity and reduced quality of life, and reports indicate that up to 44% of people over the age of 80 years may become affected. Statistically, HR is the second most common first MTPJ condition after hallux valgus (HAV), yet individuals with HR report a higher level of pain and dysfunction compared to those with HAV [1-3].

The clinical characteristics of HR are pain and reduced first MTPJ range of motion. Crepitus is often noted upon passive joint movement in severe cases [1,2]. Diagnosis is established through a combination of clinical and radiographic evaluations [2].

In most cases, the symptoms of HR are managed via conservative measures, including physical therapy, orthotics, rigid shoes, non-steroidal anti-inflammatory drugs (NSAIDs), intra-articular injections and activity modification [4]. In some cases non-operative treatments fail, at which time surgery may be considered. The technical and philosophical approaches towards the appropriate surgery for HR are vast and vary significantly [5].

There are many surgical options available in the treatment of HR, which may be broadly grouped into two categories: *joint salvage* and *joint destructive* procedures [6]. *Joint salvage* procedures include cheilectomy and chondroplasty, various metatarsal and phalangeal osteotomies, and arthroplasty procedures, including soft tissue interposition [7-11]. The aim of these procedures is to reduce pain, increase range of motion, maintain plantarflexion power and ground purchase of the hallux, and maintain stability of the first MTPJ whilst retaining the length of the first metatarsal to reduce the chance of transfer metatarsalgia [12].

*Joint destructive* procedures include resection arthroplasties, joint replacements and arthrodesis [13,14]. Arthrodesis is unique as the primary aim is pain relief without preservation of motion. It has been described as a preferred technique; however, the loss of motion has been cited as a concern [13,15,16].

Cheilectomy involves the resection of the dorsal osteophytes overlying the first MTPJ. A recent retrospective study involving 80 participants (93 feet) who underwent chielectomy with a 9.6-year follow-up found 97% of feet had good to excellent results and 92% reported pain relief and improved function [15,16].

We report a case of a 42-year-old female with painful HR who required operative treatment yet specifically requested an alternative procedure to fusion. We performed a radical cheilectomy involving the resection of the dorsal osteophytes overlying the first MTPJ with complete remodelling of the first metatarsal head and the base of the proximal phalanx. This allowed an increase of intraoperative dorsiflexion to 90 degrees. The patient reported a cessation of pain and return to her sporting activities and court-style shoes at her 12-week discharge appointment.

## Case presentation

A 42-year-old female fashion industry manager was referred to our surgical clinic regarding the progressively worsening chronic pain, joint stiffness and long-term shoe-fitting difficulties associated with her right HR pathology. Her medical history was unremarkable, taking Ventolin 100 mcg/actuation two puffs every four hours as required for mild asthma and Allegron (nortriptyline hydrochloride) 25 mg 1.5 tabs in the evenings for clinical depression. She reported multiple sensitivities (egg whites, kidney beans, almonds and feline fur). She would also experience mild pruritus and oedema from contact with latex.

She had first noticed the flexibility of her first MTPJ reducing around 2010 but her forefoot pain had only proven intolerable since 2016. Her HR symptoms had also been progressively limiting her ambulatory activities and had necessitated restrictions of her footwear selection to capacious low-heeled shoes. Her first MTPJ pain had been predominantly emanating from the dorsal articular margin of the joint without any metatarso-sesamoidal discomfort noted. A clicking sensation had been present within the joint but this sensation had eased off in recent times. She also reported experiencing episodic severe joint locking in of the first MTPJ which typically resolved with self-mobilisation and massage of the joint. This episodic articular locking had been consistently exacerbated with stair climbing as well as occurring with certain shoe styles.

Clinically, the right first MTPJ available dorsiflexion was 9 degrees and plantarflexion was 55 degrees. No rubour was exhibited on the overlying skin; however, the joint exhibited significant periarticular thickening. Pain was elicited with palpation of the dorsal articular margin and upon passive mobilisation. No pain was elicited with palpation of the metatarso-sesamoidal articulation.

**Figure 1:**
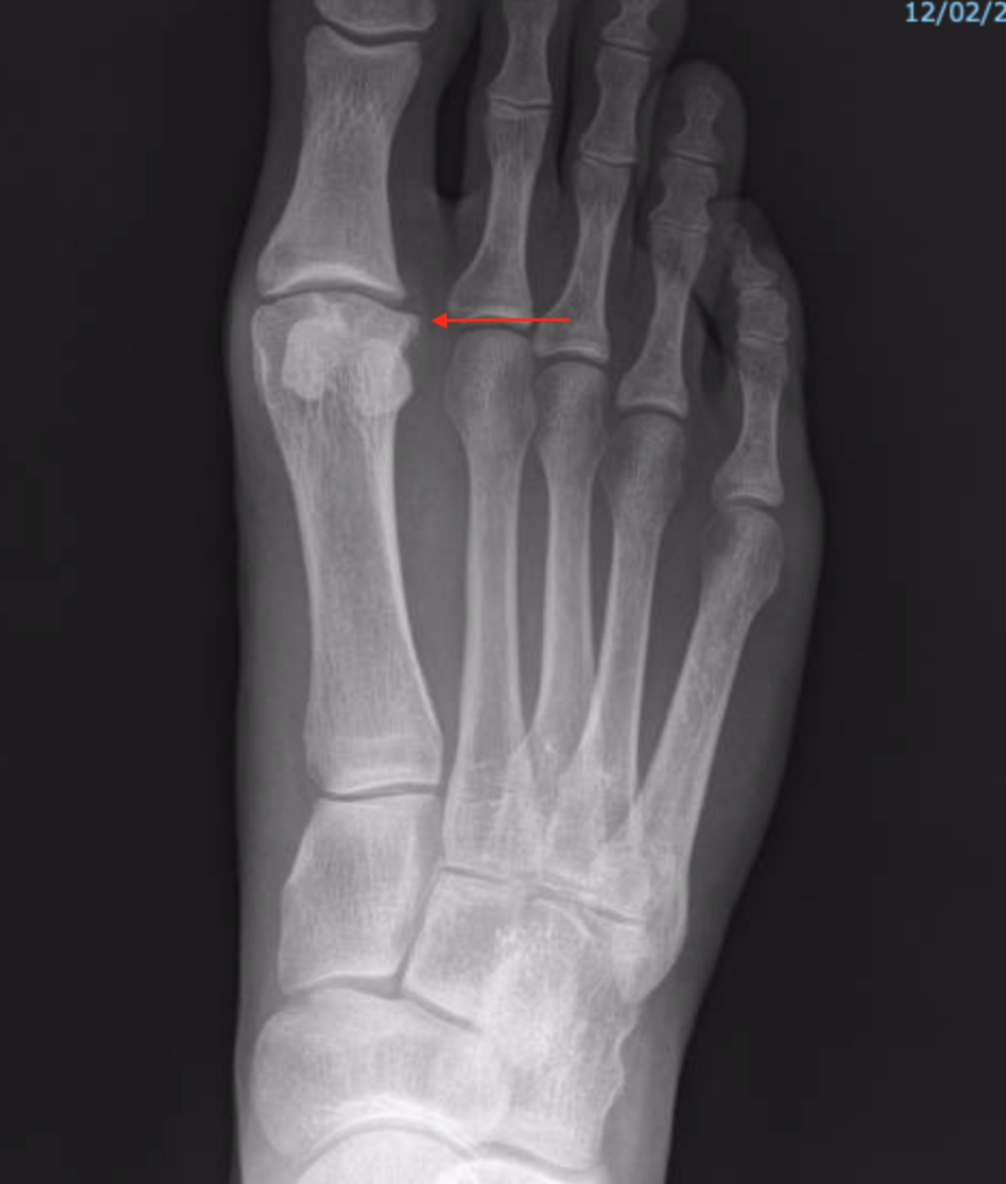
Anterior-posterior preoperative x-rays showing moderate degenerative changes of the first metatarsophalangeal joint (MTPJ).

**Figure 2:**
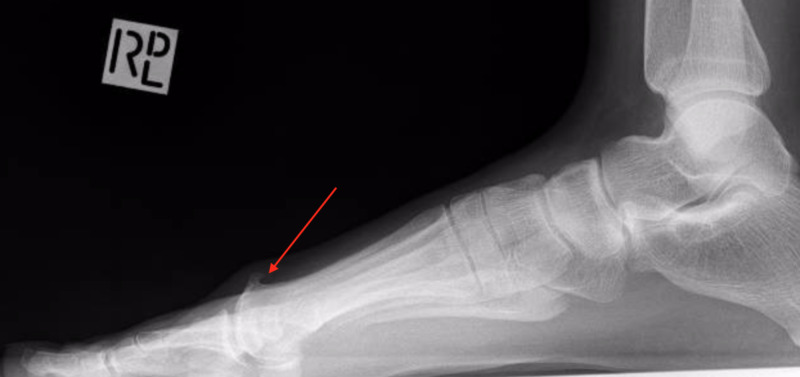
Lateral x-ray showing the dorsal osteophyte formation overlying the head of the first metatarsal.

Anterior-posterior (Figure 1) and lateral (Figure 2) projectional radiographs (X-rays) exhibited moderate right first MTPJ degenerative joint changes with a hallux abductus angle of 15 degrees and a sesamoid position of 4.

**Figure 3:**
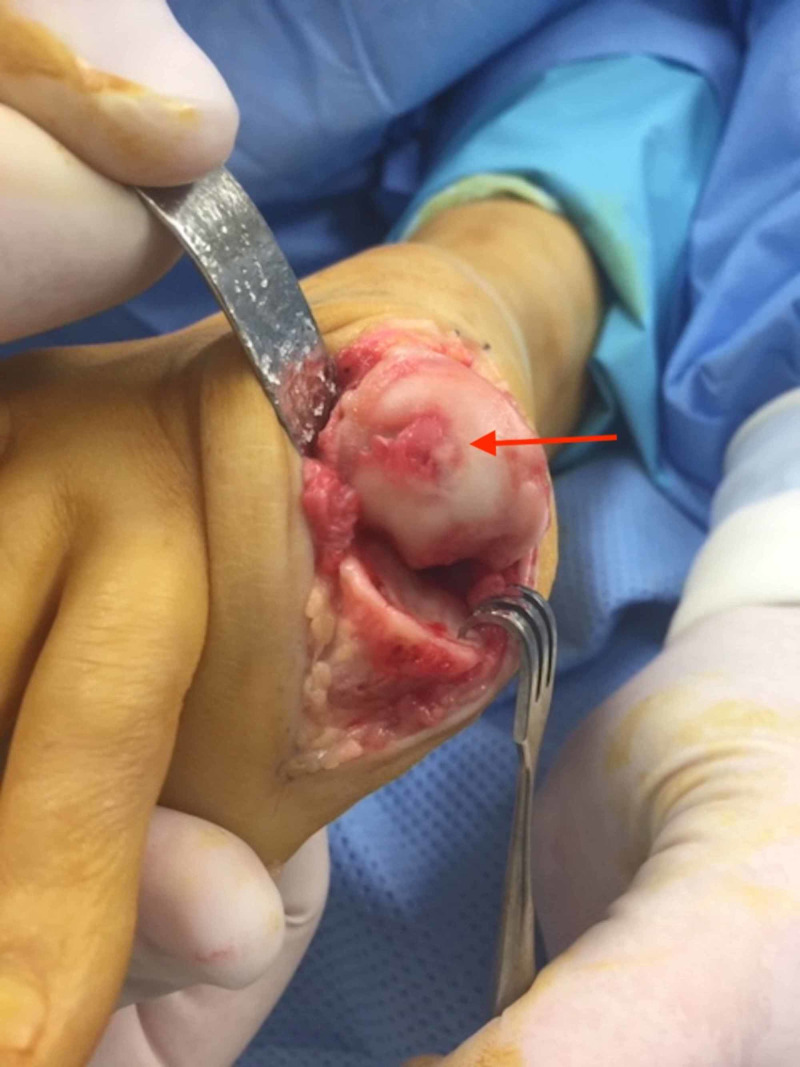
A large osteochondral defect was discovered on the head of the first metatarsal.

**Figure 4:**
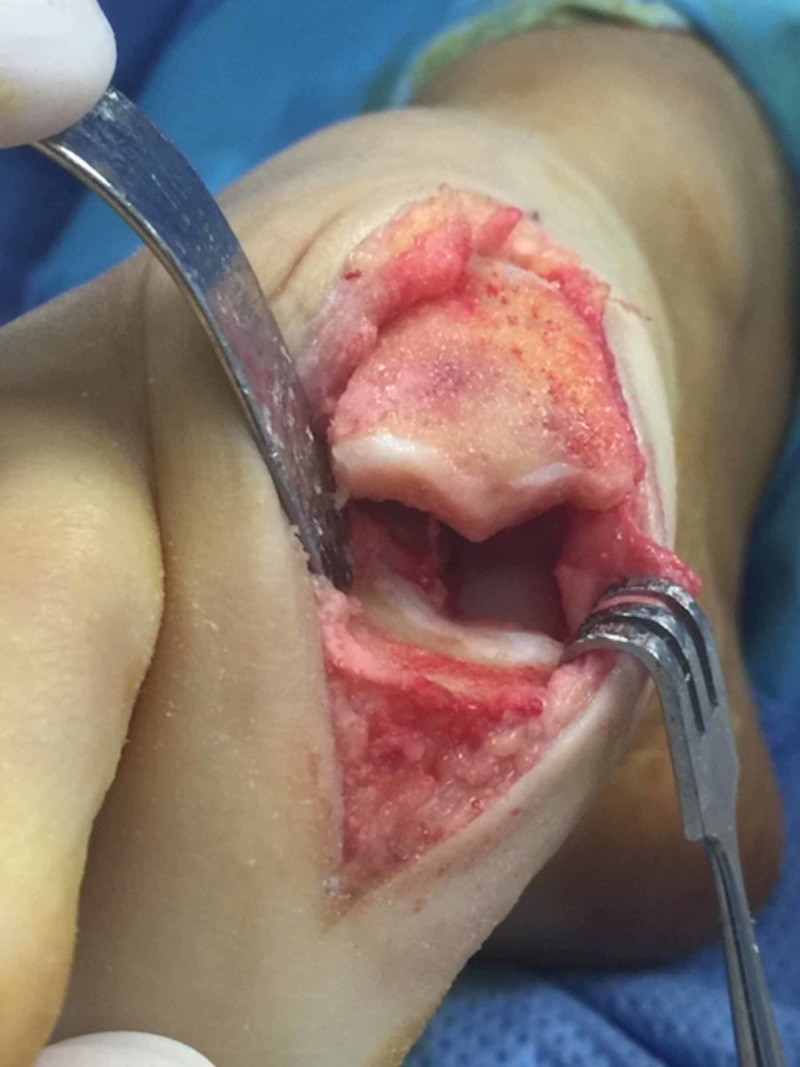
Coronal view of the radical cheilectomy showing the extent of the metatarsal head remodelling.

**Figure 5:**
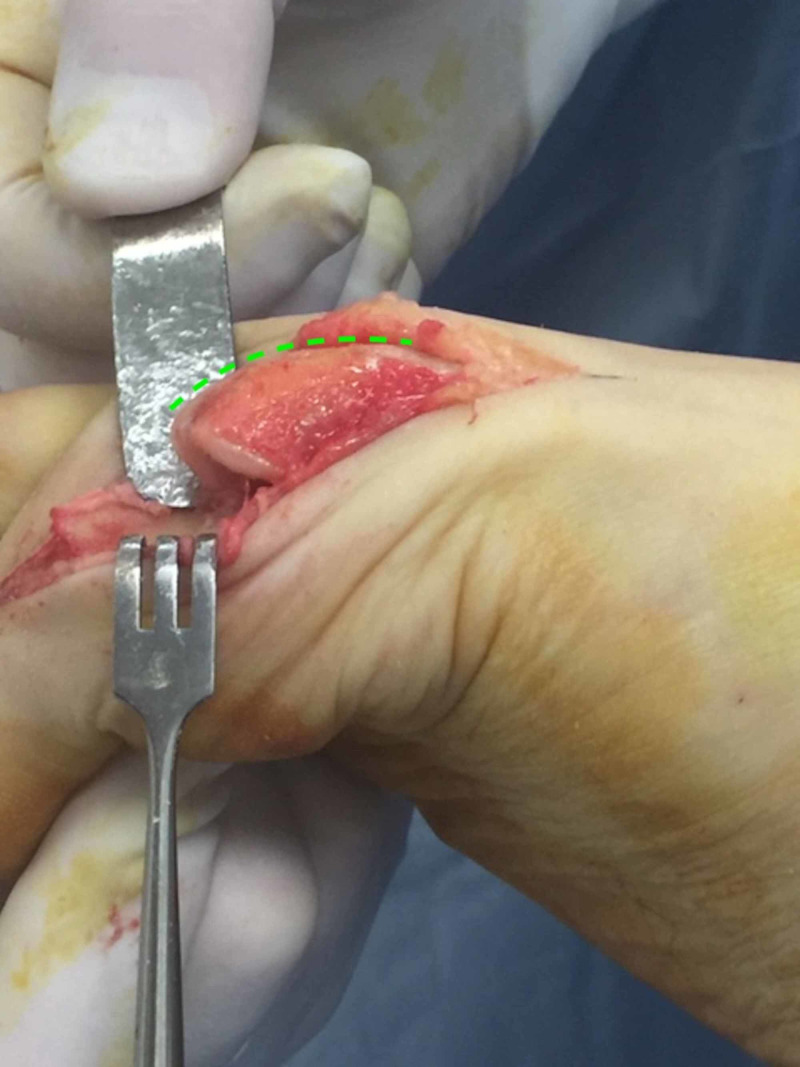
Lateral view showing the extent of the radical metatarsal head remodelling. The green line shows the parabola required to facilitate smooth joint motion and 90 degrees of dorsiflexion.

The patient was positioned supine on the operating table. After general anaesthetia was achieved, the right foot and leg were draped in the typical fashion to facilitate a sterile field. A 15 mL 0.75% ropivacaine hydrochloride/1 mL (4 mg) dexamethasone sodium phosphate injection first metatarsal Mayo block was administered. A dorso-medial longitudinal incision followed by anatomic dissection and haemostasis was used to access the first MTPJ. Severe chondromalacia and a full-thickness osteochondral defect were noted on the articular surface of the first metatarsal head (Figure 3). A power burr was employed to perform a radical dorsal, lateral and medial exostectomy (cheilectomy) to the metatarsal head (Figures 4, 5), and a modified Valenti osteotomy was performed to the base of the hallucal proximal phalanx until 90 degrees of dorsiflexion and smooth first MTPJ range of motion was achieved.

Layered wound closure was performed with a 2/0 Vicryl capsulorrhaphy and skin closure with a 3/0 running subcuticular suture. The foot was then dressed with modified-Jones compression dressings and fitted with a Darco postoperative shoe.

**Figure 6:**
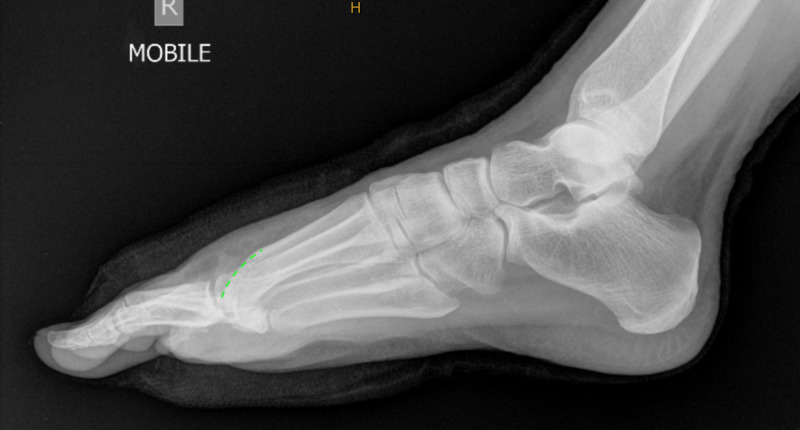
Immediate postoperative lateral radiograph illustrating the remodelled metatarsal head parabola.

**Figure 7:**
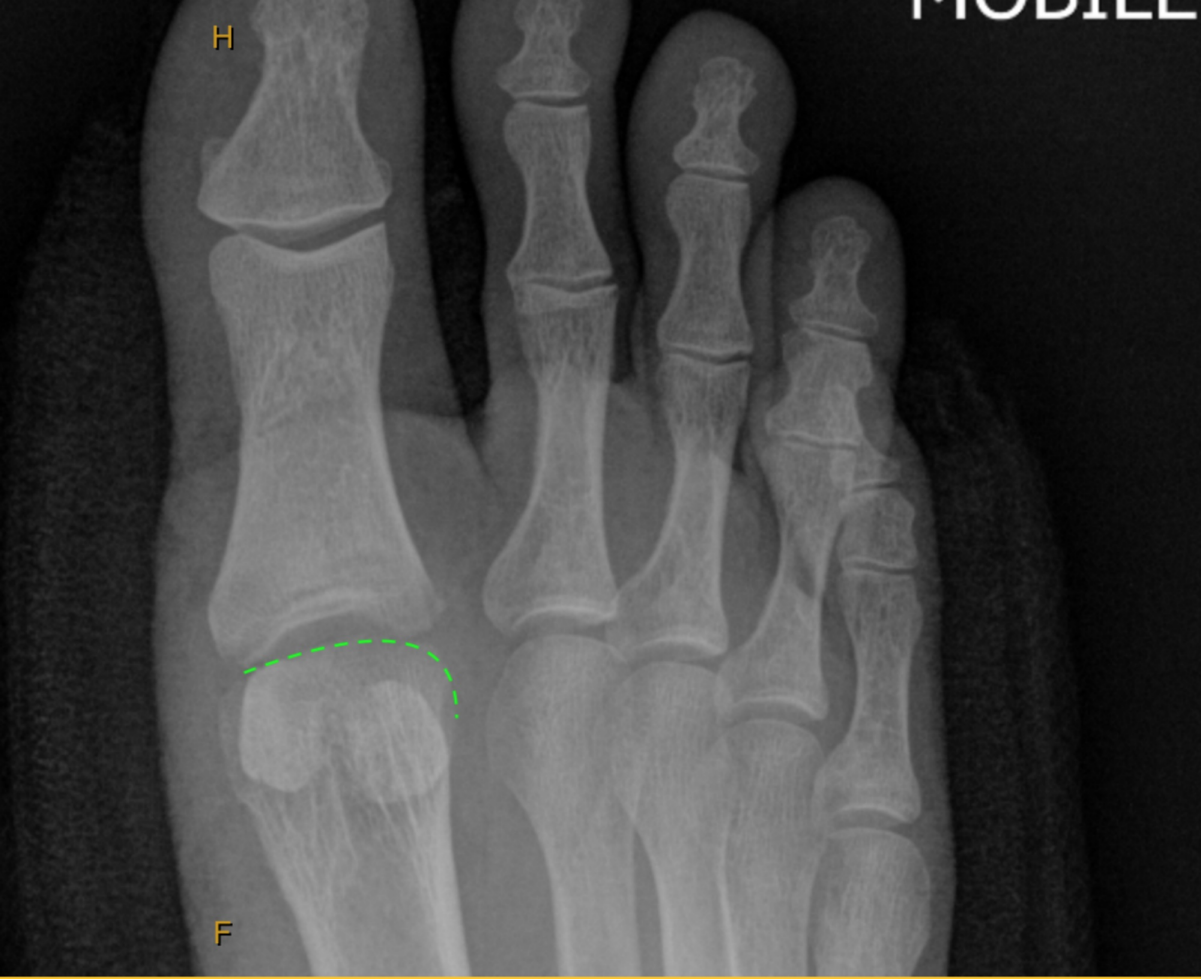
Immediate postoperative anterior/posterior radiograph showing normal metatarsal head morphology when compared to the preoperative image.

Immediate postoperative radiographs were ordered. The lateral radiograph (Figure 6) illustrated the remodelled metatarsal head parabola allowing 90 degrees of dorsiflexion. The anterior/posterior radiograph (Figure 7) showed normal anatomic contours when compared to the preoperative image.

She was allowed to weight-bear immediately and remained in her postoperative shoe for a period of three weeks before returning to capacious athletic shoes until her residual postoperative forefoot oedema settled.

## Discussion

We report a case of HR in a patient who specifically requested a joint preservation procedure rather than an arthrodesis. Her first MTPJ was surgically preserved via a modified version of a traditional cheilectomy procedure that involved increased resection of the dorsal osteophytes and chondromalacia to facilitate up to 90 degrees of dorsiflexion. This should theoretically reduce the chance of dorsal osteophyte reformation and hallux limitus over time.

This approach was undertaken for several reasons. The authors have observed in recent time a body of patients with painful HR who specifically decline the option of arthrodesis for shoe-fitting and lifestyle reasons. There are multiple joint preserving options for this patient cohort, each with their benefits and shortcomings. Some options, such as a capsular interposition arthroplasty, appear beneficial; however, the incidence of reduced joint range of motion and slight hallux flexus were cited as a concern.

Although the increased amount of osseous resection may appear profound and promote joint instability, this has not been our experience clinically. Soft tissue attachments are generally kept in situ in this approach and after capsular closure the joint is inspected for capsular weakness. Occasionally, a lateral capsulorrhaphy with two throws of 2/0 Vicryl is performed to re-enforce the lateral aspect of the joint. We have also not experienced clicking or discomfort at the plantar-distal prominence of the first metatarsal head. The proximal hallucal phalanx appears to glide smoothly along the new metatarsal head parabola as long as care and attention is taken in the bone remodelling stage of this procedure.

In the literature, Easley et al. reported a 90% subjective satisfaction rate with a mean follow-up of 63 months and residual discomfort in only 13% of feet at the final follow-up [17]. Feltham et al. reported that 91% of patients reported that they felt better than they did prior to surgery, with 78% satisfied [18]. A literature review undertaken by Maffulli et al. reported a 74% average success rate after cheilectomy surgery [19].

Sidon et al. hypothesised that the beneficial results of traditional cheilectomy were less than previously reported in the literature [20]. They investigated the functional outcomes and survival of traditional cheilectomy for the treatment of HR over sequential years for a minimum of five years postoperatively. They found that patient satisfaction following traditional cheilectomy was only 69% with a failure rate (recurrence of pain following surgery) occurring in 29% of patients, which was inconsistent with previous reports in the literature of a 90% success rate. In their study, 8% of patients transitioned to first MTPJ arthrodesis, and 58% having some residual first MTPJ pain.

The increased amount of resection was performed in light of reports in the literature of the return of the dorsal osteophytes in up to 30% of patients, which is in line with our experiences and those of other studies showing similar results of arthritic deterioration and osteophyte recurrence [19,20]. The regression to arthrosis following traditional cheilectomy appeared to occur along similar time frames to non-operative treatment [20].

## Conclusions

We report a case of HR normally amenable to arthrodesis treated with a variation to a traditional cheilectomy procedure involving a radical amount of bone resection and remodelling. This approach facilitated decreased pain and improved joint range of motion up until the patients discharge appointment at 12 weeks postoperatively. Despite the amount of bone resection, we believe that a radical cheilectomy may be an appropriate treatment option for patients who require HR surgery yet who wish not to undergo a joint destructive procedure. Anecdotally, the primary surgeon reports conversion to arthrodesis rates of less than 1% following this procedure; however, further research regarding long-term outcomes is needed. Radical cheilectomy may be an appropriate surgical treatment option for HR in patients who do not wish to undergo arthrodesis.
